# A Geographic Analysis about the Spatiotemporal Pattern of Breast Cancer in Hangzhou from 2008 to 2012

**DOI:** 10.1371/journal.pone.0147866

**Published:** 2016-01-25

**Authors:** Xufeng Fei, Zhaohan Lou, George Christakos, Qingmin Liu, Yanjun Ren, Jiaping Wu

**Affiliations:** 1 College of Environmental and Resource Sciences, Zhejiang University, Hangzhou, China; 2 Institute of Islands and Coastal Ecosystems, Zhejiang University, Zhoushan, China; 3 Hangzhou Center for Disease Control and Prevention, Hangzhou, China; Fudan University, CHINA

## Abstract

**Background:**

Breast cancer (BC) is the most common female malignant tumor. Previous studies have suggested a big incidence disparity among different cities in China. The present work selected a typical city, Hangzhou, to study BC incidence disparity within the city.

**Methods:**

Totally, 8784 female breast cancer cases were obtained from the Hangzhou Center for Disease Control and Prevention during the period 2008–2012. Analysis of Variance and Poisson Regression were the statistical tools implemented to compare incidence disparity in the space-time domain (reference group: township residents during 2008, area: subdistrict, town, and township, time frame: 2008–2012), space-time scan statistics was employed to detect significant spatiotemporal clusters of BC compared to the null hypothesis that the probability of cases diagnosed at a particular location was equal to the probability of cases diagnosed in the whole study area. Geographical Information System (GIS) was used to generate BC spatial distribution and cluster maps at the township level.

**Results:**

The subdistrict populations were found to have the highest and most stable BC incidence. Although town and township populations had a relatively low incidence, it displayed a significant increasing trend from 2008 to 2012. The BC incidence distribution was spatially heterogeneous and clustered with a trend-surface from the southwest low area to the northeast high area. High clusters were located in the northeastern Hangzhou area, whereas low clusters were observed in the southwestern area during the time considered.

**Conclusions:**

Better healthcare service and lifestyle changes may be responsible for the increasing BC incidence observed in towns and townships. One high incidence cluster (Linping subdistrict) and two low incidence clusters (middle Hangzhou) were detected. The low clusters may be attributable mainly to developmental level disparity, whereas the high cluster could be associated with other risk factors, such as environmental pollution.

## Introduction

Breast cancer (BC) is the most common malignant tumor in women, and the main cause of death among women cancer patients [1]. BC accounts for about 29% of cancer morbidity and 15% of cancer mortality among women in the United States [2] and for about 27% of cancers in European women [3]. China had a relative low BC incidence (about 30/100,000) during the period 2005–2009 [4]. However, unlike the developed countries whose incidence keeps decreasing, the incidence in China, especially in Chinese rural regions, the BC incidence showed an increasing annual percentage change (APC) of about 8.55. This increase has now become an important public health issue in China, which means that more attention needs to be paid on BC control and prevention [5].

BC has complicated pathogenic factors, such as body mass index (BMI) [6], diet [7], alcohol consumption [8], genetic susceptibility [9] and reproductive behavior [10]. Among these risk factors, socioeconomic status (SES), which could be seen as a proxy of lifestyle, as well as diet and reproductive behavior consistently showed a positive relationship with BC risk [10–12]. The observed geographic heterogeneity of both the local environmental exposure and SES [13] has been linked to the geographic disparity of BC incidence detected worldwide [14]. Ecological epidemiology studies about the geographical distribution of BC incidence could provide valuable clues concerning cancer pathology, insights regarding cancer preventive strategies and recommendations for medical sources allocation [5].

**Fig 1:**
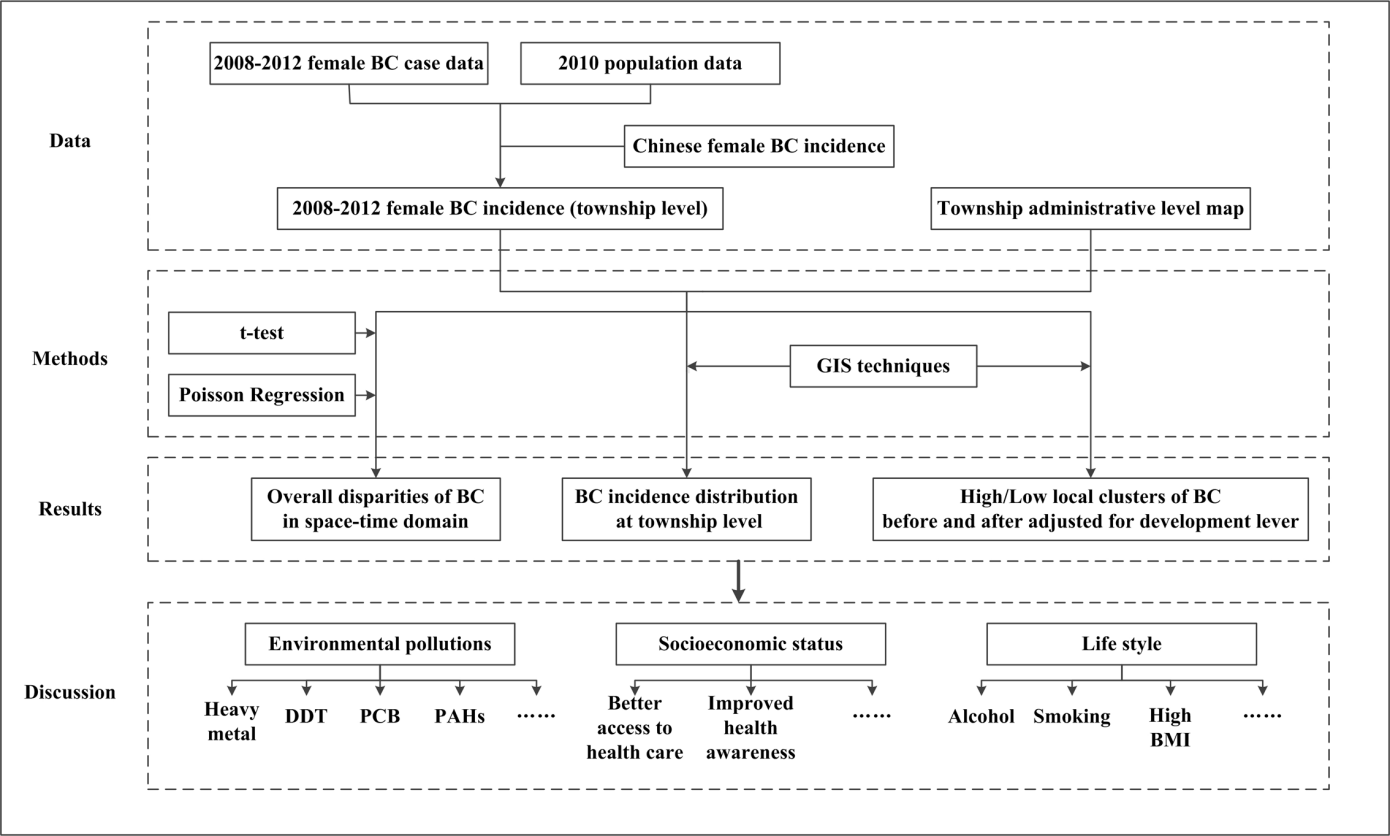
Flow chart of the study approach (BC in Hangzhou, 2008–2012).

Previous studies have emphasized the geographical heterogeneity of BC at the city level due to urban-rural and SES disparities in China [5]. In the present work we selected a typical city, Hangzhou, to study the geographical disparity of BC incidence at a finer spatial scale (township administrative level), which highlights the local effects that may influence BC risk. Specifically, the aims of the present work are to (1) investigate the temporal trend of BC cancer incidence in Hangzhou during 2008–2012, (2) analyze the spatiotemporal BC heterogeneity at the township level, (3) detect high and low BC risk clusters in the composite space-time domain, and (4) assess whether developmental level differences were responsible for these clusters. An outline of the research approach is shown in Fig 1.

## Material and Methods

### Study area

**Fig 2:**
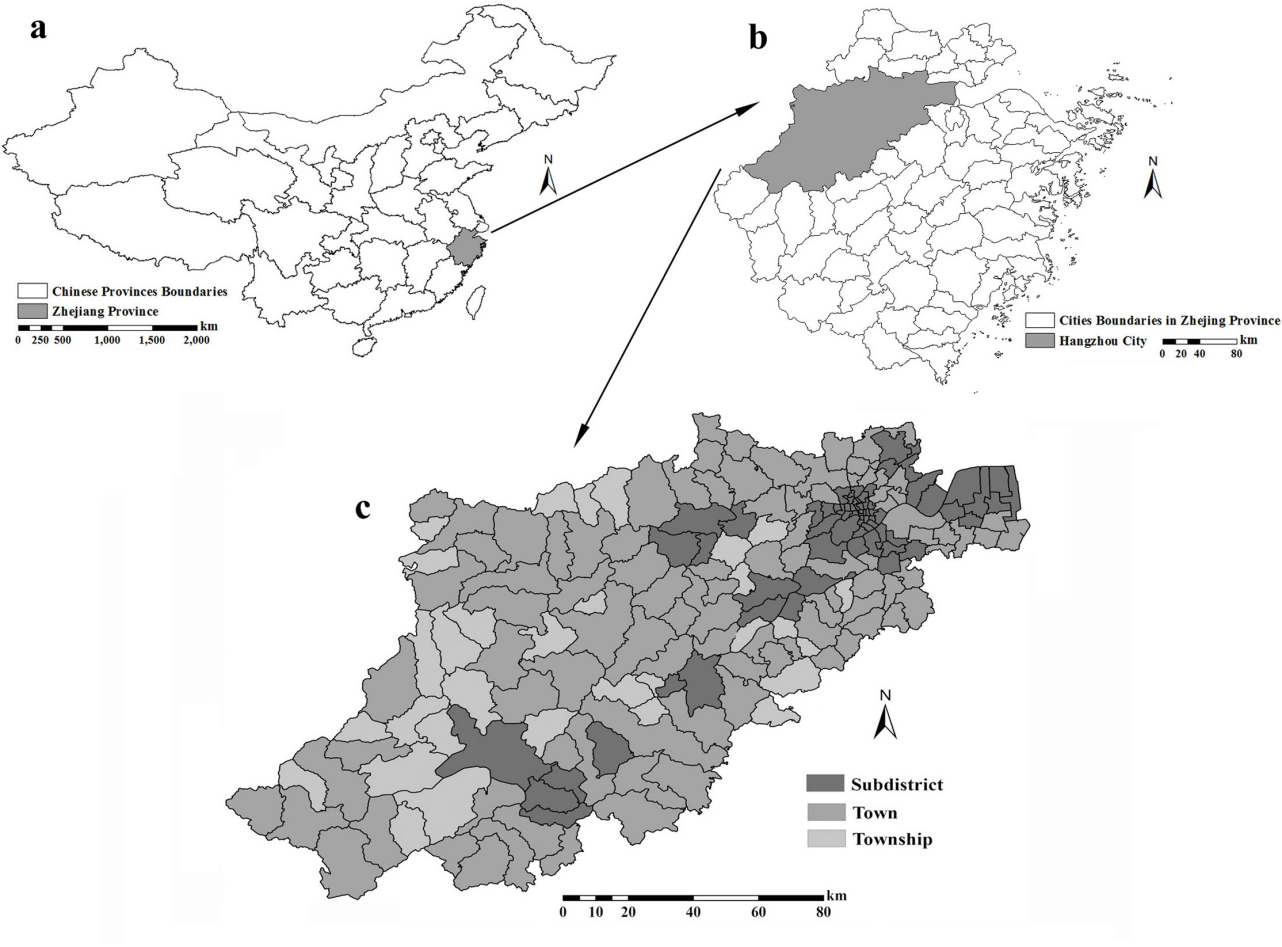
Study area and administrative division of Hangzhou. (a) Chinese province distribution map, (b) City distribution map of Zhejiang province and (c) Administrative division of Hangzhou.

Hangzhou, the capital city of Zhejiang Province (E 118°21′-120°30′, N 29°11′-30°33′), is located in the north area of Zhejiang and the southeastern coastal area of China (Fig 2) with a total area of about 16,596 square kilometers and a total household registration population of about 6.78 million. The southwestern area of Hangzhou belongs to the western hilly areas of Zhejiang Province with relative high elevation, whereas the northeastern area belongs to the north plains area of Zhejiang. Hangzhou has a typical subtropical climate with a hot and humid summer, cold and dry winter. The annual mean temperature is 17.8°C, the relative mean humidity is 70.3%, and the annual mean precipitation is 1454mm.

According to the Hangzhou administrative map obtained from the Hangzhou Bureau of Surveying and Mapping, there are 72 subdistricts, 97 towns and 31 townships in the study area (Fig 2). Subdistrict, town and township are the smallest aggregated administrative unit at which case and population data can be obtained in Hangzhou city. According to the classification criterion of the National Bureau of Statistics of the People’s Republic of China, the subdistrict area (division code: 330100) is defined as the most developed area of Hangzhou, with the highest SES and the highest education level; the township area (division code: 330102) is defined as the least developed area; and the area with development level between subdistrict and township is classified as the town area (division code: 330101). Hence, the administrative division above represents the development level, which can be seen as a proxy for SES status.

### Data

**Fig 3:**
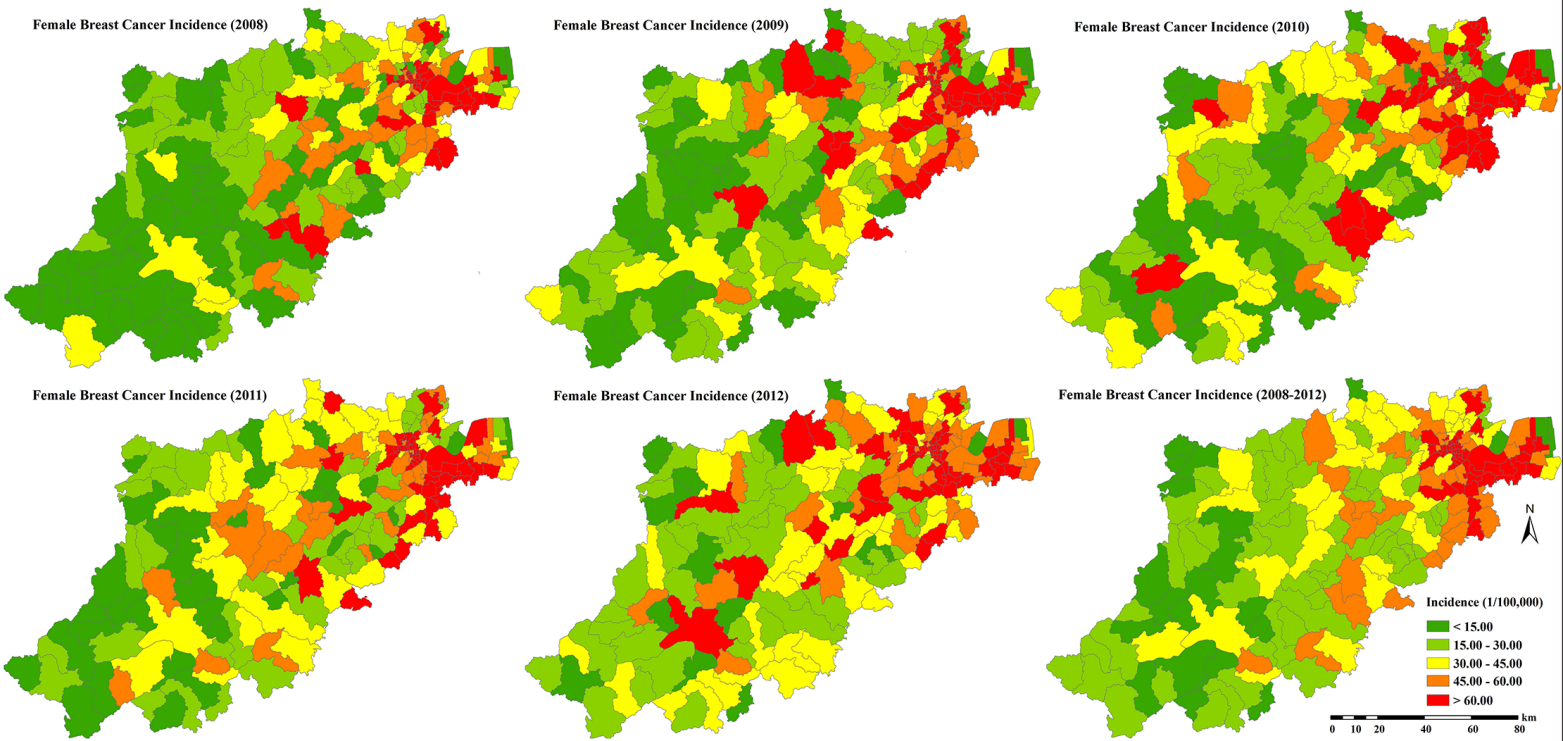
The distribution of female BC incidence in Hangzhou from 2008 to 2012 (each individual year and 5-year average).

This is an ecological epidemiology study. Patient records were anonymized by the staff of Cancer Registry Center prior to analysis. Five years of newly diagnosed BC data is obtained from the Hangzhou Center for Disease Control and Prevention (CDC). Cancer patient information in Hangzhou is registered and managed through the International Association of Cancer Registries (IACR) recommended software CanReg4. Being a monitored city of the Chinese National Cancer Center, the completeness and reliability of cancer data in Hangzhou city were checked and evaluated by the Chinese National Cancer Center (original data could be seen in the supplement data (S1 Table)). In total, 8784 female invasive breast cancer cases were confirmed between 2008 and 2012 (International Classification of Disease: ICD-10 code C50). Specifically, 1643, 1727, 1820, 1812 and 1782 cases were confirmed during the years 2008, 2009, 2010, 2011 and 2012, respectively. All the patients were located at specific administrative units (72 subdistricts, 97 towns and 31 townships) according to their detailed residence information. Since age information about the BC cases is not readily available in Hangzhou (that could be used, e.g., in the Poisson and scan statistics techniques), age-standardized incidence per 100,000 residents was computed by means of an indirect method [15]. This method used female age-specific population data at the township, town and sub-district levels of Hangzhou (obtained from the Hangzhou Public Security Bureau, PSB) combined with the most recent female BC incidence of different age categories over China (available by the Chinese National Cancer Center, [16]), which was used as the reference group. The indirect age-standardization method can control for differences due to heterogeneous age structure at the township levels. The distributions of BC incidence during each year together with the 5-year BC average are shown in Fig 3.

### Analysis methods

In this work, the analysis consisted of both a methodological and a computational part, as follows.

#### Methodologically

(*i*) the Analysis of Variance (ANOVA) technique was employed to compare BC incidence disparity between populations living in different areas (subdistrict, town, and township) during the period 2008–2012. When testing for linear trend, the models were fitted by the mean values of each category and continuous category ranking (the rank 0, 1, 2, 3 and 4 was assigned to the year 2008, 2009, 2010, 2011 and 2012, respectively). Poisson regression modeling, which is a classical method of comparing BC incidence among populations, was implemented to study the mean and the 95% confidence interval (CI) of BC relative risk (RR) of populations living in different areas and during different years [17]. The regression parameter exponent is the BC incidence RR that was compared to the reference group (i.e., people living in a township area during 2008). An RR value > 1 or < 1 denotes, respectively, a higher or a lower risk of BC development. Linear trend testing was conducted as described above.

(*ii*) Space-time scan statistics was implemented to analyze spatiotemporal high and low BC clusters, assuming that the cancer cases follow a Poisson distribution [18, 19]. This assumption was justified following the consideration of several candidate probability models, including the normal (continuous data form), exponential (survival time data), ordinal (ordered categorical data), multinomial (categorical data), Poisson, Bernoulli and space-time permutation (count data) models. Considering our data form, the last three models could be possibly used in our analysis. Yet, the space-time permutation model is used to detect case number clusters due either to an increased disease risk or to different geographical population distributions at different times, whereas the Bernoulli model needs population data in every year. As we only have 2010 population data, the Poisson model was selected as the best choice, which is a model that could be used to adjust for development levels. The probability of a case being diagnosed at a particular location was compared to the null hypothesis that the probability of cases diagnosed at a particular location was equal to the probability of cases diagnosed in the whole study area. The scan time frame was set at 90% of the study period, and it also included purely spatial clusters (i.e., the whole study period) in order to utilize the available information during the entire study period. The maximum spatial window was primarily set at 50% of the total population, and then it was set at 25% and 10% of the total population to detect the influence of different spatial windows. As there was no big difference between different spatial windows, except for small spatial windows with more small continuous clusters, the medium spatial window (25%) was selected to present the cluster analysis results. Cluster significance was evaluated by comparing the likelihood of the specified cluster to the likelihoods of the 9,999 Monte Carlo simulations.

(*iii*) Geographical Information System (GIS) techniques were used to represent spatial variation in terms of spatial distribution and cluster maps of BC incidence.

Computationally:

(*i*) the ANOVA and the Poisson regression model were conducted by Stata 12.1 [20].

(*ii*) the space-time scan statistics was performed by means of the SaTScan software [17], and

(*iii*) BC incidence and the cluster maps were generated by the ArcMap 9.3 [21]. All the *P* values and 95% CI were 2-sided and the significance threshold was set at 0.05.

## Results

A relatively stable BC incidence was observed in Hangzhou during the period 2008–2012. The overall incidence was 48.81, 51.30, 54.06, 53.83 and 52.94 per 100,000 individuals for 2008, 2009, 2010, 2011 and 2012, respectively. However, there exist big differences between regions. The subdistrict area with the highest incidence rate showed little BC disparity among the 5 years considered, whereas the township area with the lowest incidence rate expressed a significant trend increase (*P* = 0.040). BC incidence increased from 13.45/100,000 in 2008 to 25.66/100,000 in 2012, with an APC of about 17.5. The town area with medium incidence rate also showed a significant trend increase (*P* = 0.044), from 32.31/100,000 in 2008 to 41.58/100,000 in 2012, with an APC of about 6.5 (Table 1).

**Table 1:** Female BC incidence in Hangzhou, 2008–2012.

Time	Subdistrict	Town	Township	*P*1^a^	*P*2	*P*3
**2008**	60.80^b^	32.31	13.54	<0.01**	<0.01**	<0.01**
	(50.27–71.94) ^b^	(27.66–36.74)	(6.91–21.95)			
**2009**	60.55	36.66	19.75	0.01**	<0.01**	<0.01**
	(52.79–68.36)	(31.14–41.90)	(11.24–30.06)			
**2010**	61.57	38.34	22.09	<0.01**	<0.01**	<0.01**
	(52.96–69.81)	(32.99–43.60)	(13.92–30.41)			
**2011**	65.55	37.04	18.81	<0.01**	<0.01**	<0.01**
	(57.96–73.15)	(31.98–41.91)	(11.36–26.26)			
**2012**	62.04	41.58	25.66	<0.01**	<0.01**	<0.01**
	(55.35–68.73)	(37.06–46.43)	(21.08–30.66)			
***P* for trend**	0.299	0.040*	0.044*			

^a^ *P*1: ANOVA for incidence disparity between subdistrict and town areas, *P*2: ANOVA for incidence disparity between town and township areas and *P*3: ANOVA for incidence disparity between subdistrict and township areas.

^b^ Incidence and 95% confidence interval (per 100,000 individuals)

*Significant at 0.05 level

**Significant at 0.01 level

The Poisson Regression results showed that people living in town (RR = 2.03, 95% CI = 1.75–2.35) and subdistrict (RR = 3.18, 95% CI = 2.74–3.68) areas have a higher risk to develop BC. The linear trend was significant at the 0.05 level. The years 2010–2012 exhibited a slightly higher BC risk compared to the year 2008. The corresponding RRs were 1.05 (95%CI = 0.98–1.12), 1.11 (95%CI = 1.04–1.18), 1.10 (95%CI = 1.03–1.18) and 1.08 (95%CI = 1.01–1.16) for the years 2009, 2010, 2011 and 2012, respectively. However, no significant temporal linear trend was observed (Table 2).

**Table 2:** Poisson Regression for female BC in Hangzhou, 2008–2012.

Characteristics	RR^a^	95%CI^b^	*P* for trend
**Administrative level**			
**Township**	Ref^c^		
**Town**	2.03	[1.75–2.35] **	
**Subdistrict**	3.18	[2.74–3.68] **	0.02*
**Time**			
**2008**	Ref		
**2009**	1.05	[0.98–1.12]	
**2010**	1.11	[1.04–1.18] **	
**2011**	1.10	[1.03–1.18] **	
**2012**	1.08	[1.01–1.16] **	0.146

^a^ Relative Risk, calculated as exp (*β*_*1*_) based on Poisson regression model

^b^ 95% confidence interval

^c^ Reference Category

*Significant at 0.05 level

**Significant at 0.01 level

The spatial distribution of BC incidence exhibited a clustered pattern. Moran’s I values were 0.353, 0.345, 0.352, 0.422, 0.380, and 0.563 for the years 2008, 2009, 2010, 2011, 2012, and the 5-year averaged BC incidence, respectively. The BC incidence maps (Fig 3) demonstrated that the incidence distribution was spatially heterogeneous across the study area. The northeast part of Hangzhou showed higher BC incidence, whereas lower BC incidence was observed mainly in the southwest part of Hangzhou. This geographical disparity was more evident at the scale of the 5-year averaged BC incidence map.

**Fig 4:**
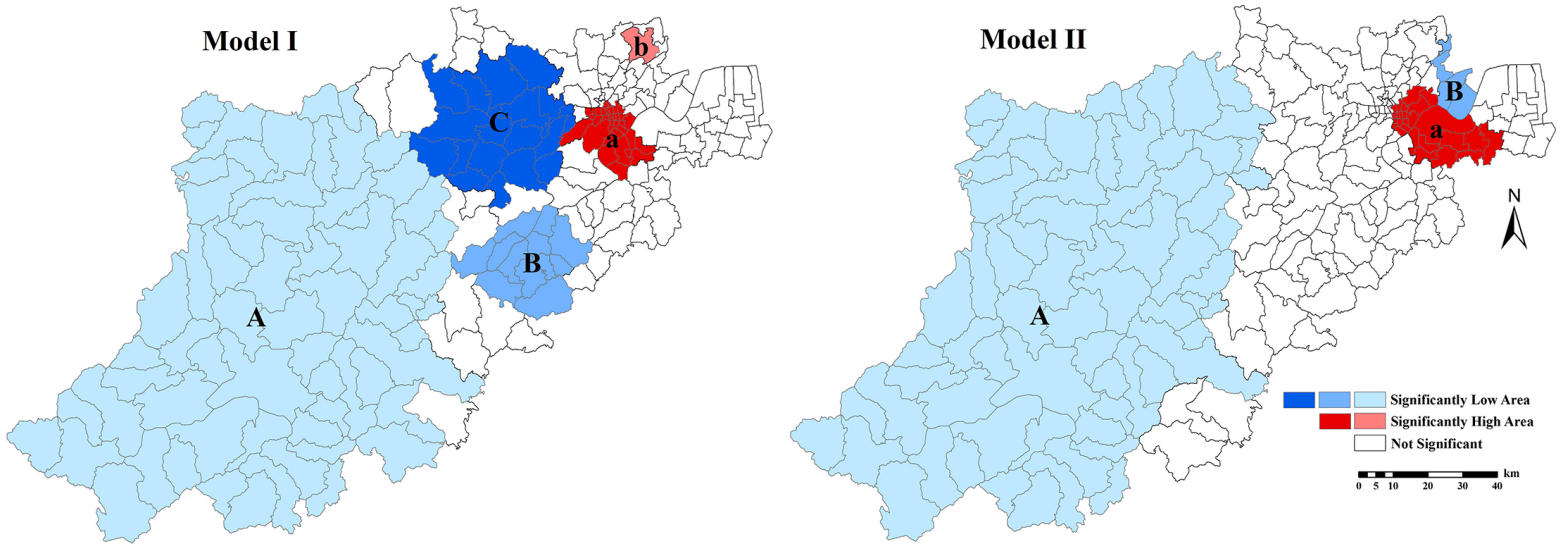
Spatiotemporal clusters of female BC in Hangzhou from 2008 to 2012. Model I: no adjustment. Model II: adjusted for development level.

Two significant high clusters of BC incidence were detected by means of space-time scan statistics (no adjustment).One cluster was located at the northeast downtown area of Hangzhou (2008–2012 time frame, RR = 1.89), the other was the Linping subdistrict (2008–2012 time frame, RR = 2.49). Three significant low BC clusters were found in the middle and southwest area of Hangzhou: A (2008–2012 time frame, RR = 0.42), B (2008–2011 time frame, RR = 0.51), and C (2008–2011 time frame, RR = 0.69) (Fig 4 Model I). Detailed information about these high/low clusters is given in Table 3. The clusters pattern detected by the space-time scan statistics technique was similar to that of other clusters detection methods (Getis-Ord Gi*, (S1 Fig), Local Indicators of spatial association, (S2 Fig), and Anselin Local Moran I, (S3 Fig)). BC clusters did not differ significantly among the various methods considered, thus offering a high level of confidence to this distribution pattern. The one high cluster (Linping subdistrict) and the two low clusters (B and C) detected in Model I were no longer significant when additionally adjusted for development level (Fig 4, Model II). Therefore, the disparity between development levels may be responsible for the high/low BC incidence observed in these areas. The most significant high cluster ‘a’ and low cluster ‘A’ detected in Model I showed little difference when additionally adjusted the development level in Model II, and a new significant low cluster appeared in the north area of Hangzhou (Fig 4, Model II). Detailed information about the clusters detected by Model II is given in Table 4.

**Table 3:** Space-time scan statistics of female BC in Hangzhou, 2008–2012.

	Time-frame	Population	Observed^a^	Expected^b^	RR^c^	*P*^d^
**Significant high clusters**						
**a**	2008–2012	805,525	3,278	2,101	1.89	<0.01**
**b**	2008–2012	27,918	144	58	2.49	<0.01**
**Significant low clusters**						<0.01**
**A**	2008–2012	713,722	888	1,862	0.42	<0.01**
**B**	2008–2011	134,053	183	349	0.51	<0.01**
**C**	2008–2011	278,956	410	582	0.69	<0.01**

^a^ Observed Number of female breast cancer cases

^b^ Expected Number of female breast cancer cases

^c^ Relative Risk

^d^ Significance test from 9999 Monte Carlo Simulations

**Significant at 0.01 level

**Table 4:** Space-time scan statistics of female BC in Hangzhou (adjusted for administrative level), 2008–2012.

	Time-frame	Population	Observed^a^	Expected^b^	RR^c^	*P*^d^
**Significant high clusters**						
**a**	2008–2012	725,128	3,006	2,146	1.61	<0.01**
**Significant low clusters**						<0.01**
**A**	2008–2012	784,572	1,047	1,734	0.55	<0.01**
**B**	2008–2012	103,437	100	339	0.29	<0.01**

^a^ Observed Number of female breast cancer cases

^b^ Expected Number of female breast cancer cases

^c^ Relative Risk

^d^ Significance test from 9999 Monte Carlo Simulations

**Significant at 0.01 level

## Discussion

Our study found that, overall, the BC in Hangzhou exhibited a relative stable incidence during the period 2008–2012. Although people living in the subdistrict (highest incidence) area showed little BC incidence differences during the years 2008 to 2012, people living in town and township areas showed a significant incidence increase, with an APC of 6.5 and 17.5 at the town and township areas, respectively. The distribution of BC incidence is spatially heterogeneous and clustered with a trend-surface from the southwest low incidence area to the northeast high incidence area (this was particularly valid for the 5-year averaged BC incidence). Using space-time scan statistics, significant high clusters were detected in the northeast downtown area of Hangzhou and significant low clusters were detected in the middle and southwest area of Hangzhou. When additionally adjusted for administrative unit (development level), which could be seen as a proxy for SES status, one significant high cluster (Linping district) and two significant low clusters in the middle of study area (clusters B and C in Model I) disappeared, suggesting that SES differences may be responsible for these high/low clusters.

Unlike the developed countries of the West, in which BC incidence keeps decreasing [3, 22], the present study found that the BC incidence was stable in the most developed area of Hangzhou, and what is worse, in the less developed Hangzhou area, the incidence showed a significantly increasing trend. Considering the relative large female population (subdistrict area: 1.61million, town area: 1.57million and township area: 0.18million) and the large number of BC cases in each area (S1 Table), the annual BC incidence and increasing trend between years in each area can be determined more rigorously than the expected incidence fluctuation [23]. This result is consistent with our previous study [5], which suggested that BC incidence remained stable in Chinese urban regions but exhibited an increasing APC of about 8.55 for Chinese rural regions. Beyond the population mobility and *Hukou* policy factors mentioned in the previous study, there may exist some other factors for the increasing BC incidence in the less developed area of Hangzhou, as follows. In recent years more resources were shifted in China toward the development of small towns, especially in big cities like Hangzhou, implying that Hangzhou town and townships are experiencing a rapid development stage [24]. People living in these areas have a higher income, better health service, and improved health awareness than before (S2 Table), which lead to more BC being diagnosed in its early stage, resulting to an increased BC incidence [25, 26]. Furthermore, economic development is usually accompanied by environmental pollution. Numerous environmental pollutants (such as Ionising radiation, heavy metals, dioxins and organic solvents discharged through industry activities) may lead to higher BC risk [27–29]. Following the rapid economic development, people living in Hangzhou town and township areas are exposed to higher environmental pollutant levels which could be a serious health concern [30]. Lastly, as has been reported in the relevant literatures with increasing income, many local people adopt a more westernized lifestyle, increased alcohol consumption [8], cigarette smoking [31] and higher BMI values [6], which may be also responsible for the observed incidence increase [32].

In China, the notion of city usually refers to a large area with heterogeneous cancer incidence and SES. In our previous study, which was conducted at city level, Hangzhou city was considered as a point datum, and the BC incidence and SES were the means over the entire Hangzhou area [5], Thus, Hangzhou was classified as a developed city with stable incidence, which means that within-city differences have not been taken into account. In this study, BC incidence distribution and SES were homogeneous at the township administrative level, and the incidence distribution was overall stable in the Hangzhou area, but in the less developed areas BC incidence distribution exhibited a significantly increasing trend and it was spatially heterogeneous. This behavior could provide a better understanding of the spatiotemporal disease pattern within the city, and it could be more helpful for local BC control and prevention than in previous studies [33]. When apparent BC clusters appeared in the study area that could not be dismissed as a stochastic phenomenon (linked to the Monte Carlo simulations of space-time scan statistics), sufficient attention should be paid to the population at risk, and immediate action should be taken to gain a better understanding of the underlying causal mechanisms at the risk areas [34].

The Moran’s I results showed that BC incidence was spatially clustered at the township level in Hangzhou during 2008–2012. This phenomenon was more apparent at the 5-year average BC incidence scale, which allows a more stable incidence estimation at the larger population base. Then, space-time scan statistics was employed to detect spatial clusters across the study area. One significant high cluster (Linping subdistrict) with the highest RR and two significant low clusters were detected in Model I (no adjustment variable was controlled). However, when additionally adjusted for development level, these high/low clusters were no longer defined as significant. In the Linping subdistrict, the economic center of Yuhang county (adjacent to Qianjiang Economic Development Zone) is experiencing a rapid development in recent years. Considering its rapid development and the scan statistics results obtained, it is reasonable to assume that the development level was responsible for Lingping’s cluster pattern. Besides, all significant clusters appeared during the entire study period, except for the low clusters B and C (2008–2011 time frame) in Model I. This phenomenon highlighted the influence of economic development on BC risk. Clusters B and C belong to the suburb area of Hangzhou, which is one of the areas experiencing the fastest economic development in recent years. With increasing development level and BC incidence, BC cases forming low clusters were no longer detected in these areas during 2012. As a proxy for SES status, development level can be a surrogate of several BC risk factors: healthcare service and mammography, reproductive behavior, lifestyles etc. [35–37]. Further studies are needed to evaluate the contributions of certain factors (healthcare service, changes in reproductive behavior, and lifestyle) to increased BC risk.

When adjusted for development level, the most significant high cluster (Fig 4, cluster a) and the most significant low cluster (Fig 4 cluster A) showed little difference, while the opposite was the case of clusters when no adjustment was controlled. Other environmental exposures (such as ionizing radiation), and organochlorines (like dichloro-diphenyl-trichloro, DDT, Polychlorinated biphenyls, PCBs, and polycyclic aromatic hydrocarbons PAHs) [38–40], which have an uneven spatial distribution across the study area, might be the explanation of the heterogeneous BC risk observed. Further studies are needed to confirm whether special environmental pollution is responsible for local BC risk, especially for the high risk metropolitan area, as it includes more than 10% of the total population in Hangzhou. A new low cluster appeared when adjusted for development level. Since there are many universities in this area, younger age structure may be the reason for this low cluster.

This ecological study has some limitations that are worth-mentioning. First, it focused on the geographical disparity of BC incidence at the population level. Hence, it is difficult to control for individual confounders, such as BMI and alcohol consumption that may affect BC incidence [8, 41]. Second, BC incidence showed a significantly increasing trend in the less developed areas, suggesting that better health service (mammography and treatment) is responsible for early stage cancer diagnosis, which, in turn, contributed to the observed incidence increase. However, due to the lack of information concerning patients at the diagnosis stage, we could not evaluate quantitatively the effect of such factors. Third, due the nature of this study (ecological design), all the results should be interpreted with caution, and further in-situ study is needed to improve the interpretation [42].

In sum, using surveillance data obtained from the Hangzhou CDC, this work found that BC incidence was significantly higher in the more developed subdistrict areas of Hangzhou, while maintaining a relatively stable level. However, in less developed areas (towns and townships) the BC incidence showed a significantly increased trend from 2008 to 2012. The spatial incidence distribution was uneven and clustered across the study area. Being a proxy for comprehensive SES index, development level is responsible for the high BC cluster in the Linping subdistrict and the low clusters in the middle area of Hangzhou. On the other hand, the development level had little influence on the significant low cluster in the southwest Hangzhou area and the significant high cluster in the northeast Hangzhou area. Lastly, other risk factors, such as environmental exposure and lifestyle, may contribute to the high RR in these areas.

## Supporting Information

S1 Fig Cluster pattern of female breast cancer incidence from 2008 to 2012, detected by Getis-Ord Gi* (each year and 5-year average). (TIF)

S2 Fig Cluster pattern of female breast cancer incidence from 2008 to 2012, detected by Local Indicators of Spatial Association (each year and 5-year average). (TIF)

S3 Fig Cluster pattern of female breast cancer incidence from 2008 to 2012, detected by Anselin Local Moran’s I (each year and 5-year average). (TIF)

S1 Table Original female breast cancer case in Hangzhou from 2008 to 2012. (XLSX)

S2 Table Economic and health data in Hangzhou. (XLSX)
